# Tourniquet use in total knee replacement surgery: a feasibility study and pilot randomised controlled trial (SAFE-TKR study)

**DOI:** 10.1136/bmjopen-2020-043564

**Published:** 2021-01-22

**Authors:** Peter David Henry Wall, Imran Ahmed, Claire Edwin, Muhamed M Farhan-Alanie, Helen Parsons, Andrew James Price, Jane Warwick, Charles E Hutchinson, Martin Underwood, Andrew Metcalfe, SAFE-TKR Study Group, B Rahman

**Affiliations:** 1 Warwick Clinical Trials Unit, University of Warwick Warwick Medical School, Coventry, UK; 2 Trauma and Orthopaedic, Warwick Clinical Trials Unit, Coventry, UK; 3 Division of Health Sciences, University of Warwick, Coventry, Warks, UK; 4 Nuffield Department of Orthopaedics, Rheumatology and Musculoskeletal Sciences, University of Oxford, Oxford, UK; 5 Warwick Medical School, University of Warwick, Coventry, UK; 6 Trauma and Orthopaedics, University Hospitals Coventry and Warwickshire NHS Trust, Coventry, UK

**Keywords:** knee, adult surgery, orthopaedic & trauma surgery

## Abstract

**Introduction:**

Tourniquets are routinely used during total knee replacement (TKR) surgery. They could increase the risk of thromboembolic events including cerebral emboli, cognitive decline, pain and other adverse events (AEs). A randomised controlled trial to assess whether tourniquet use might safely be avoided is therefore warranted but it is unclear whether such a trial would be feasible.

**Methods:**

In a single-site feasibility study and pilot randomised controlled trial, adults having a TKR were randomised to surgery with an inflated tourniquet versus a non-inflated tourniquet. Participants underwent brain MRI preoperatively and within 2 days postoperatively. We assessed cognition using the Mini-Mental State Examination (MMSE), Montreal Cognitive Assessment (MoCA) and Oxford Cognitive Screen (OCS) and thigh pain using a Visual Analogue Scale at baseline and days 1 and 2, and 1 week postsurgery. AEs related to surgery were recorded up to 12 months.

**Results:**

We randomised 53 participants (27 tourniquet inflated and 26 tourniquet not inflated). Fifty-one participants received care per-protocol (96%) and 48 (91%) were followed up at 12 months. One new ischaemic brain lesion was detected. Of the cognitive tests, MoCA was easy to summarise, sensitive to change with lower ceiling effects compared with OCS and MMSE. There was a trend towards more thigh pain (mean 49.6 SD 30.4 vs 36.2 SD 28 at day 1) and more AEs related to surgery (21 vs 9) in participants with an inflated tourniquet compared with those with a tourniquet not inflated.

**Conclusion:**

A full trial is feasible, but using MRI as a primary outcome is unlikely to be appropriate or feasible. Suitable primary outcomes would be cognition measured using MoCA, pain and AEs, all of which warrant investigation in a large multicentre trial.

**Trial registration number:**

ISRCTN20873088.

**Strengths and limitations of this study**

- Prospectively designed randomised controlled trial with prepublished protocol.
- Blinding of outcome assessors and patients in order to prevent detection and performance bias.
- Included a range of different cognitive and functional outcome measures in order to assess the feasibility of each.
- This feasibility study had lower rates of recruitment compared with previous randomised controlled trials performed in our centre.

## Introduction

More than 106 000 total knee replacements (TKR) were performed in the UK in 2018.^1 2^ Over 90% of surgeons prefer to use a tourniquet when performing TKRs.^3 4^ A thigh tourniquet applied may help reduce intraoperative bleeding and allow cement to interdigitate more effectively within the bone.^5 6^

A tourniquet which squeezes the thigh and restricts blood flow may however increase the risks of surgery including thigh pain and complications such as deep vein thrombosis (DVT) and wound infection.^3 7 8^ The incidence of symptomatic venous thromboembolism (VTE) following knee replacement surgery has been reported to be between 0.7% and 0.9%.^9 10^ Preliminary evidence suggests that thrombosis and other debris held behind a tourniquet during surgery may embolise when the tourniquet is released and in some cases reach the systemic circulation including the carotid and cerebral circulation.^11 12^Cerebral embolisation may explain the higher than expected prevalence of postoperative cognitive deficit following TKR. In published reports, this varies from 41% to 75% at 7 days to 18% to 45% at 3 months postoperatively.^13^

It is possible that a tourniquet may have an important effect on cognitive outcomes after TKR, however we cannot determine this at present as there are no randomised controlled trials (RCTs) reporting the effect of tourniquet use on cognition or the risk of cerebral emboli. The only way to determine the effect of tourniquet use on TKR outcomes is to undertake an RCT with participants randomised to surgery either with or without tourniquet.

It is unclear whether patients and surgeons would take part in a trial, or whether surgeons would adhere to randomised treatment during surgery. In addition, outcome measures of ischaemic emboli and cognition need to be trialled in this population to establish if they can be administered without ceiling effects and are sensitive to detect tourniquet-related change.

### Aims and objectives

To determine the feasibility of a full trial of tourniquet use in TKR surgery.

Coprimary objectives were to estimate recruitment, adherence to protocol and follow-up of participants to a trial.

Secondary objectives were to:

1. Evaluate MRI for detecting postoperative ischaemic cerebral emboli, including an estimate of the size and direction of any effect.
2. Evaluate tools for detecting postoperative cognitive impairment. These tools include Mini-Mental State Examination (MMSE), Montreal Cognitive Assessment (MoCA) and the Oxford Cognitive Screen (OCS).
3. Evaluate other candidate primary or secondary outcome measures for assessment within a larger trial including: thigh pain, symptomatic VTE, mortality, revision surgery, blood transfusion requirements, function and health-related quality of life.

## Methods

The study received National Research Ethics Committee (NREC) approval in January 2016 (15/WM/0455) and the study protocol was published.^14^ Eligible patients were invited to take part in a single-centre two-arm pilot randomised controlled trial at University Hospitals Coventry and Warwickshire. The eligibility criteria were as follows:

Inclusion:

1. Primary unilateral TKR.
2. Age≥18.
3. Able to provide written informed consent and to participate fully in the study procedures.

Exclusion:

1. Participants for whom MRI is contraindicated due to:
  Non-compliant defibrillator or heart pacemaker.
  Non-compliant metallic foreign body.
  Claustrophobia:
2. Tourniquet contraindicated (eg, peripheral vascular disease).
3. Previous participation in SAFE-TKR (Safety and Feasibility Evaluation of Tourniquets for Total Knee Replacement) study.

NHS research nurses recruited and obtained written consent. Participants completed baseline questionnaires and had a diffusion-weighted MRI (following a set imaging protocol) of their brain not more than 1 month prior to surgery. Participants were allocated to a trial arm on the day of surgery using a minimisation algorithm in a 1:1 ratio. The algorithm was designed to ensure balance between the treatment arms for participants with a history of VTE. Participants were blind to treatment allocation until the end of the study period (March 2019). Participants had routine primary unilateral cemented TKR surgery by one of the 13 consultant surgeons working within Trust using their standard technique. Both groups had a thigh tourniquet applied to help ensure participant blinding. Once the participant was fully anaesthetised and all the surgical drapes including a patient screen was in place one of the following interventions was applied.

### Group A (tourniquet inflated)

A pneumatic tourniquet cuff was applied and inflated. The operating surgeons used their standard tourniquet inflation pressures to achieve ‘limb occlusion’ before the initial skin incision was made and deflated once the procedure was deemed completed by the surgeon. At a minimum, this was after the components were inserted.

### Group B (tourniquet not inflated)

A pneumatic tourniquet cuff was applied but not inflated at any point during the procedure.

All participants received the following routine chemical and mechanical VTE prophylaxis as per trust guidance:

1. Intermittent pneumatic calf compression until mobility was no longer substantially reduced.
2. Low molecular weight heparin (or unfractionated heparin for people with severe renal impairment or established renal failure), started 6 hours postoperatively and continued for 14 days.

All participants had the same routine early mobilisation physiotherapy regimen unless otherwise stated by the operative surgeon.

The feasibility outcomes of this research were to estimate recruitment, adherence to protocol and follow-up of participants to a trial.

The following clinical outcomes were collected:

1. Cerebral emboli
  Patients had a further diffusion-weighted MRI of their brain within 3 days of surgery to look for evidence of new acute ischaemic brain lesions. Diffusion-weighted MRI was chosen as it is the most powerful tool for diagnosing acute ischaemic brain lesions caused by cerebral microembolism providing high level of sensitivity and specificity.^15–17^
  The MRIs were double read by two experienced consultant radiologists (KS and HM) and blinded to the treatment allocation. The total number of baseline and new acute brain lesions detected on MRI was recorded.
2. Cognition.
  The following cognitive tests were completed at baseline, day 1, day 2, and 1 week following surgery and carried out by a study team member who was blind to treatment allocation.
  - *Mini-Mental State Examination (MMSE*)
    Scores range from 0 to 30, with 24 or less defined as impaired cognition.
  - *Montreal Cognitive Assessment (MoCA*)
    Scores range from 0 to 30, with a score of 25 or lower was defined as mild cognitive impairment.
  - *Oxford Cognitive Screen (OCS*)
    Returns a visual snapshot of a person’s cognitive profile which summarises performance on five cognitive domains (attention and executive function, language, memory, number processing and praxis).
3. Thigh pain.
  A visual analogue scale (0 mm being no pain and 100 mm being the worst pain imaginable) was used to record thigh pain at baseline, day 1, day 2 and 1 week.
  Oxford Knee Score (OKS) collected preoperatively and at 1 week, 6 and 12 months postoperatively. The score ranges from 0 to 48, where 48 represents the best outcome and 0 represents the worst outcome.
4. Knee pain and function.
5. Health-related quality of life.
  EuroQol-5D (EQ-5D-5L) scores preoperatively and at 1 week, 6 and 12 months postoperatively.
6. Blood loss.
  - *Number of intraoperative/postoperative blood transfusions until discharge*.
  - *Change in haemoglobin concentration* (*Hb g/L*).
7. Adverse events related to surgery.

Participant questionnaires and healthcare records collected adverse events (AEs) up to 12 months postoperatively. Two blinded doctors (IA and CE) determined whether AEs should be classified as related to the surgery and whether they should be classified as serious adverse events (SAEs).

### Assessment and blinding

It was not possible to blind the clinicians administering the intervention. Participants and researchers who collected outcome measures were blinded to treatment allocation.

### Follow-up

Participants with any outstanding postoperative cognitive tests (typically either day 2 or 1 week) after discharge from hospital had these collected by a trained researcher visiting them at home. Questionnaires were administered by post or email at each time point.

### Sample size and statistical analysis

As this was primarily a feasibility study and not designed to measure effect, a formal sample size calculation was not performed. The a priori plan was to recruit and obtain clinical outcome data for 50 patients to enable good estimates of recruitment, protocol adherence, follow-up and SD for the continuous outcome measures (including the cognitive tests).^18^

Percentages for recruitment, protocol adherence and follow were prepared and displayed in a Consolidated Standards of Reporting Trials (CONSORT) diagram with reasons for missing data detailed.

Standard descriptive statistics (eg, medians and ranges or means and variances, dependent on the distribution of the outcome). Baseline data were summarised to highlight any characteristic differences between allocation groups. As this was a feasibility study and pilot RCT, statistical comparisons were not performed, and p-values were not generated.

## Results

Between April 2017 and February 2018, we screened 451 patients in clinics and at preoperative education classes and of these 422 were eligible. However, of the 422 eligible patients, 324 were not approached for recruitment, for 239 (74%) this was due to a lack of capacity to deliver the postoperative MRI within 3 days of surgery. A further 85 (26%) were eligible but the surgeon in charge of care declined to take part in the trial. The surgeons felt they were not in equipoise and had a strong preference to use a tourniquet for all cases.

**Figure 1:**
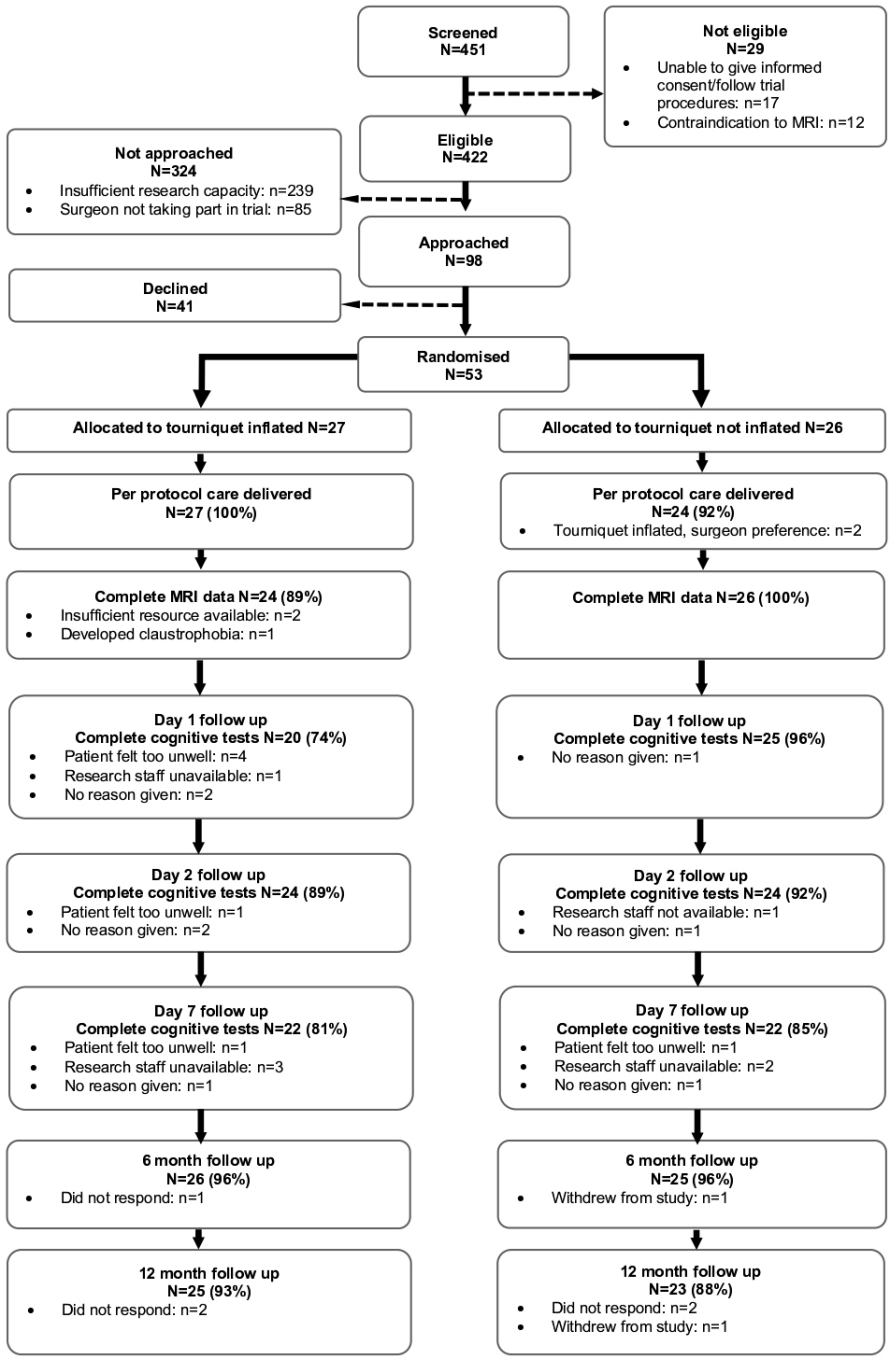
CONSORT diagram demonstrating recruitment and follow-up rates in addition to reasons for loss to follow-up. CONSORT, Consolidated Standards of Reporting Trials.

Ninety-eight people were approached for recruitment, of which we randomised 53 (54%). Fifty-one participants (96%) received treatment per protocol. Two participants did not have their treatment per-protocol, having been allocated to tourniquet not inflated they had surgery with a tourniquet inflated. These two participant crossovers in treatment were due to surgeon preference at the time of surgery and not in response to excessive intraoperative bleeding. Five participants were lost to follow-up at 1 year. Participant flow through the study is illustrated in the CONSORT diagram (figure 1).

Baseline characteristics of participants are presented in table 1. There were small imbalances between the groups, which is to be expected given the small size of the study. Mostly, these were not thought to be clinically meaningful except that two participants in the tourniquet inflated group had a previous history of VTE compared with one in the tourniquet not inflated group. Five participants allocated to tourniquet inflated were already on anticoagulant medication and six allocated to tourniquet not inflated.

**Table 1:** Baseline characteristics of randomised participants

	Tourniquet inflated, n=27	Tourniquet not inflated, n=26	All participants, n=53
Background information available (n, %)	25 (93)	24 (92)	49 (93)
Study knee (left; n, %)	14 (56)	12 (50)	26 (53)
Age at study registration (in years; mean, SD)	69.4 (6.9)	67.5 (6.8)	69.4 (6.9)
Sex (female; n, %)	11 (41)	13 (50)	24 (45)
BMI (kg/m^2^)			
Mean, SD	30.5 (5.5)	30.6 (5.2)	30.6 (5.3)
Missing	1 (4)	1 (4)	2 (4)
Current smoker (yes; n, %)	1 (4)	0	1 (2)
Weekly alcohol consumption (n, %)			
0	9 (36)	12 (50)	21 (43)
1–7	11 (44)	7 (29)	18 (37)
8–14	3 (12)	1 (4)	4 (8)
15–21	1 (4)	2 (8)	3 (6)
More than 21	1 (4)	1 (4)	2 (4)
Missing	0	1 (4)	1 (2)
Previous DVT, PE or VTE (yes; n, %)	2 (8)	1 (4)	3 (6)
Previous cerebrovascular accident/stroke (n, %)			
No	25 (100)	22 (92)	47 (96)
Yes	0	1 (4)	1 (2)
Missing	0	1 (4)	1 (2)
Taking blood thinning medication (n, %)			
No	20 (80)	17 (71)	37 (76)
Yes	5 (20)	6 (25)	11 (22)
Missing	0	1 (4)	1 (2)
Previous surgery/injury to study knee (yes, n, %)	12 (48)	12 (50)	24 (49)
Hospital location (n, %)			
University Hospital	3 (11)	4 (15)	7 (13)
Rugby St Cross	24 (89)	22 (85)	46 (87)
Baseline MRI lesion category (n, % per group)			
None	3 (12.5)	3 (11.5)	6 (11)
Less than 10 lesions	8 (33.3)	9 (34.6)	17 (32)
10–30 lesions	6 (25.0)	8 (30.8)	14 (26)
Over 10 lesions with significant atrophy	7 (29.2)	4 (15.4)	11 (21)
Over 30 lesions with significant atrophy	0	2 (7.7)	2 (4)
Missing	3 (11%)	0	3 (6)

BMI, body mass index; DVT, deep vein thrombosis; PE, Pulmonary Embolism; SVT, symptomatic venous thromboembolism.

### Clinical outcomes

1. Cerebral emboli.
  Fifty participants had baseline and day 2 MRI brain images for analysis. Three participants did not have a postoperative MRI, all of these had an inflated tourniquet; two were due to inadequate resource availability and one because the patient became claustrophobic and the scan was abandoned. There was only one participant in which the postoperative MRI demonstrated a change from baseline. In this MRI one new ischaemic brain lesion was detected. The lesion detected was on one slice (slice thickness 4 mm) and measured 15 mm^2^. This participant did not have a tourniquet inflated and had fewer than 10 lesions on their baseline MRI.
2. Cognition.

### Mini-Mental State Examination

**Figure 2:**
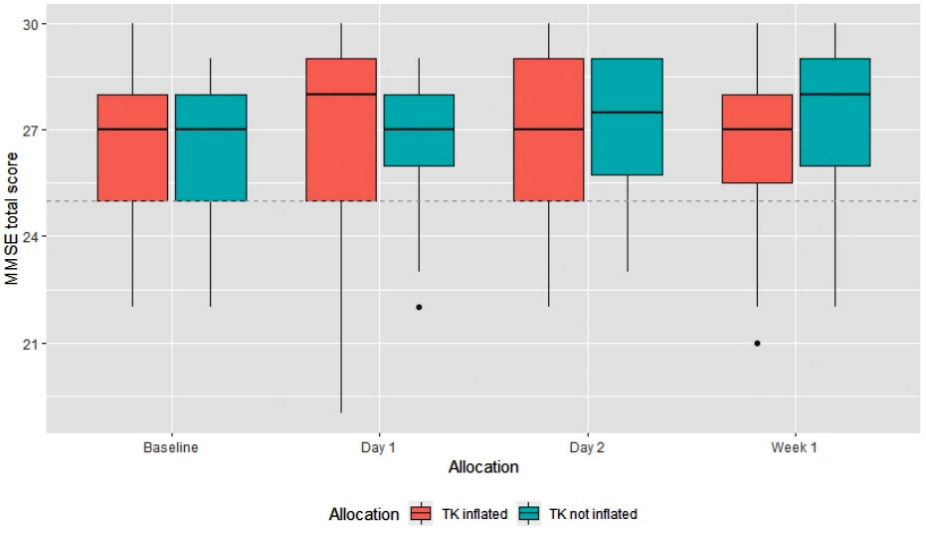
Box plot of MMSE total score by allocation group and time point. *Each box represents the first and third quartiles of the data, black middle line represents median value. Whiskers cover the max or minimum value or 1.5 times the interquartile range (IQR). Values outside 1.5*IQR are plotted individually as dots. MMSE, Mini-Mental State Examination.

MMSE scores were broadly the same at baseline in both groups; mean 26 in the tourniquet inflated group and mean 26.5 in the tourniquet not inflated group (see table 2). The mean scores in both groups remained between 26 and 27, SD <3 from day 1 to 1 week. The lowest proportion of normal scores (70%) and maximum variability (SD: 2.8) was apparent at day 1 in the group with tourniquet inflated. However, at day 1, data completion was only 78% and was missing for six participants in the tourniquet group. Four of these six participants did not complete the cognitive tests because they felt too unwell and for two, the reason for the missing data was not recorded. This was in comparison to data completion of 96% in the tourniquet not inflated group with missing data for one participant, with the reason for the missing data not recorded. Figure 2 is a box plot of MMSE scores between the two groups.

**Table 2:** MMSE scores

Score	Baseline (mean, SD)		Day 1 (mean, SD)		Day 2 (mean, SD)		Week 1 (mean, SD)	
	T inflated	T not inflated	T inflated	T not inflated	T inflated	T not inflated	T inflated	T not inflated
Scores present (n,%)	26 (96)	25 (96)	21 (78)	25 (96)	25 (93)	24 (92)	23 (85)	23 (88)
Total score (mean, SD)	26.8 (2.2)	26.5 (2.2)	26.8 (2.8)	26.5 (1.9)	26.8 (2.2)	27 (2.1)	26.4 (2.2)	27.7 (2.1)
Normal score (total ≥25) (n,%)	23 (85)	19 (73)	19 (70)	20 (77)	21 (78)	20 (77)	20 (74)	22 (85)
Scored maximum score (n, %)	3 (11)	0 (0)	2 (7)	0 (0)	2 (7)	0 (0)	1 (4)	4 (15)

MMSE scores range from 0 to 30, with 24 or less defined as impaired cognition.

MMSE, Mini-Mental Score Examination; T, tourniquet.

### Montreal Cognitive Assessment

**Figure 3:**
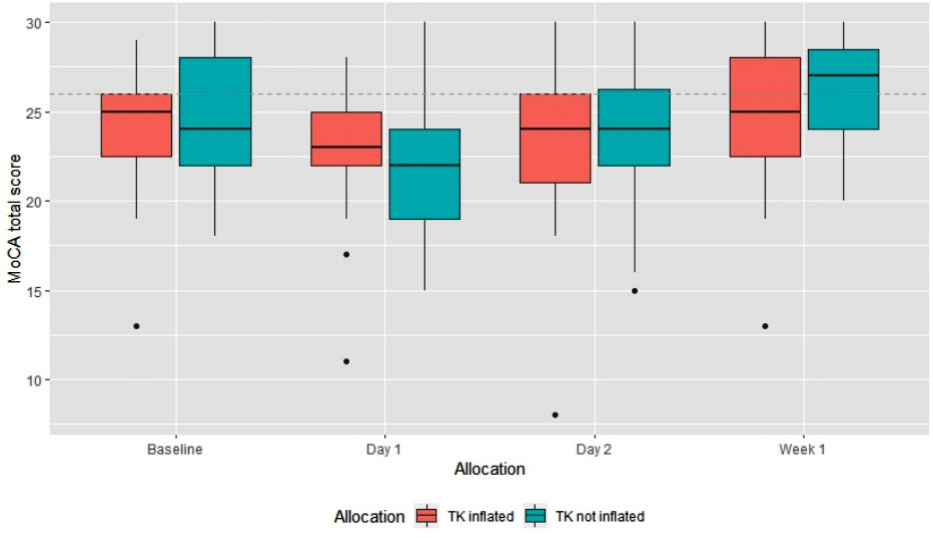
MoCA total score by allocation group and time point. MoCA, Montreal Cognitive Assessment.

MoCA scores at baseline were broadly similar between groups; mean 24.2 SD 3.6 in the group allocated to tourniquet inflated and 24.4 SD 3.5 in the group with tourniquet not inflated. In both groups, there was a deterioration in cognition at day 1 (mean 22.7 SD 3.7 in the tourniquet inflated group and 22 SD 4 in the tourniquet not inflated group) with 11% having normal scores in the tourniquet inflated group and 19% in the tourniquet not inflated group. However, data were missing for six participants in the group allocated to tourniquet, four of these participants felt too unwell to complete the test and for two participants, the reason was not recorded. In comparison at day 1, data were missing for one participant in the group allocated to tourniquet not inflated, the reason for this missing data was not recorded. In both groups, cognition improved on day 2 and was broadly back to the baseline level at 1 week (mean 24.6 SD 4 in the tourniquet inflated group and 26 SD 3 in the tourniquet not inflated group). MoCA scores are presented in table 3 with box plots in figure 3.

**Table 3:** MoCA scores

Score	Baseline (mean, SD)		Day 1 (mean, SD)		Day 2 (mean, SD)		Week 1 (mean, SD)	
	T inflated	T not inflated	T inflated	T not inflated	T inflated	T not inflated	T inflated	T not inflated
Scores present (n, %)	27 (100)	25 (96)	21 (78)	25 (96)	25 (93)	24 (92)	23 (85)	23 (88)
Total score (mean, SD)	24.2 (3.6)	24.4 (3.5)	22.7 (3.7)	22 (4)	23 (4.4)	23.7 (3.6)	24.6 (4)	26 (3)
Normal score (total ≥26; n, %)	12 (44)	11 (42)	3 (11)	5 (19)	7 (26)	8 (31)	10 (37)	14 (54)
Scored maximum (n, %)	0	0	0	1 (4)	1 (4)	1 (4)	1 (4)	2 (8)

Scores range from 0 to 30, with a score of 25 or lower was defined as mild cognitive impairment.

MoCA, Montreal Cognitive Assessment; T, tourniquet.

### OCS data

Further details on OCS data can be viewed in online supplemental file 1.

10.1136/bmjopen-2020-043564.supp1 Supplementary data

### Thigh pain

Thigh pain scores are shown in table 4. At baseline, mean pain scores were lower in the group allocated to tourniquet inflated (12.9 SD 20.9) compared with the tourniquet not inflated group (20.5 SD 22.7). At days 1 and 2, both groups had higher mean pain scores than baseline, but this was higher in the group allocated to tourniquet inflated (49.6 and 32.8, respectively) compared with the tourniquet not inflated group (36.2 and 28.4, respectively). At 1 week, mean pain scores were still higher than baseline but the difference between groups was less (39.3 SD 33.1 in the tourniquet inflated group and 38.5 SD 29.9 in the tourniquet not inflated group).

**Table 4:** Thigh pain, OKS and EQ-5D outcomes based on intervention group. data provided as mean and SD

Outcome	Time point	N valid responses (inflated, not inflated)	Tourniquet inflated (mean, SD)	Tourniquet not inflated (mean, SD)	All participants (mean, SD)
Thigh pain VAS	Baseline	27, 26	12.9 (20.9)	20.5 (22.7)	16.6 (21.9)
	Day 1	23, 25	49.6 (30.4)	36.2 (28.0)	42.6 (29.6)
	Day 2	25, 27	32.8 (28.3)	28.4 (26.8)	30.6 (27.4)
	Week 1	25, 26	39.3 (33.1)	38.5 (29.9)	38.9 (31.2)
OKS	Baseline	26, 26	21.0 (9.8)	19.0 (6.5)	20.0 (8.3)
	Week 1	23, 24	17.8 (8.4)	18.5 (6.7)	18.1 (7.5)
	6 months	25, 25	35.5 (7.1)	29.9 (10.1)	32.7 (9.2)
	12 months	24, 23	37.0 (8.3)	33.7 (10.1)	35.4 (9.3)
EQ-5D	Baseline	27, 26	0.434 (0.258)	0.459 (0.208)	0.446 (0.233)
	Week 1	25, 25	0.308 (0.325)	0.400 (0.241)	0.353 (0.287)
	6 months	26, 25	0.731 (0.173)	0.683 (0.183)	0.707 (0.178)
	12 months	25, 23	0.736 (0.207)	0.729 (0.219)	0.733 (0.211)

EQ-5D, EuroQol-5D; OKS, Oxford Knee Score; VAS, Visual Analogue Scale.

### Knee pain and function

OKS scores are shown in table 4. At baseline, mean OKS scores were higher in the group allocated to tourniquet inflated (21 SD 9.8) compared with the tourniquet not inflated group (19 SD 6.5). At 1 week, both groups had lower mean OKS scores than baseline. The mean scores then recovered and were higher than baseline in both groups by 6 and 12 months.

### Health-related quality of life

EQ-5D scores are shown in table 4. At baseline, mean EQ-5D scores were lower in the group allocated to tourniquet inflated (0.434 SD 0.258) compared with the tourniquet not inflated group (0.459 SD 0.208). At 1 week, both groups had lower mean EQ-5D scores than baseline. The mean scores then recovered and were higher than baseline in both groups by 6 and 12 months.

### Blood loss

No patients in either group received a blood transfusion postoperatively. The mean change in haemoglobin concentration between baseline and prior to hospital discharge was 23.6 (SD 8.5) in the group with tourniquet not inflated and 17.8 (7.5) in the group with tourniquet inflated.

### Adverse events related to surgery

All AEs reported and deemed related to the surgery are shown in table 5. All of the AEs met the criteria to be classified as SAEs. There were no deaths reported in the study period. There was a trend to more AEs related to surgery in the group allocated to tourniquet inflated compared with the group with tourniquet not inflated.

**Table 5:** Adverse events related to surgery by group

Adverse event	Tourniquet inflated	Tourniquet not inflated
DVT	2	0
Wound infection	3	2
Urinary retention	7	2
Hyponatraemia	2	1
Chest infection	2	1
Ileus	1	0
Acute kidney injury	1	0
Further procedures		
MUA	0	1
Aspiration	2	1
Wound washout	0	1
Revision (DAIR)	1	0
Total	21	9

DAIR, debridement and implant retention; DVT, deep vein thrombosis; MUA, manipulation under anaesthetic.

## Discussion

### Feasibility study outcomes

We have demonstrated that it is feasible to recruit and follow-up patients in a trial comparing TKR with a tourniquet versus TKR without a tourniquet. Adherence to randomisation was good with just two participants not having treatment per protocol due to surgeon preference. The pilot obtained a follow-up rate greater than 83% for all the outcome measures and time points. Only one surgeon expressed a strong preference not to take part (preferred to use a tourniquet) indicating that equipoise within the community would be sufficient to complete a larger trial.

One of the main challenges in recruiting to the pilot trial was obtaining sufficient research capacity to perform postoperative MRI scans with 57% of eligible potential participants not being recruited for this reason. Recruitment (54%) in the pilot RCT was also low compared with similar surgical trials in our unit.^19^ The main reason for this was that people were reluctant to have MRI scans. Hence, a larger trial using ischaemic brain lesions detected on MRI as a primary outcome may be difficult.

We trialled three cognitive tests to determine which was the most suitable in a full trial. MMSE and MoCA both had single summary measures which were easier to interpret, with high completion rates for both (>85% at all time points). However, MoCA had evidence of less ceiling effects (up to 8% at any time point in a group had a maximum score) compared with MMSE (up to 15% at any time point had a maximum score). Our data also suggest that MoCA is more sensitive for detecting cognitive impairment in this patient population with a higher percentage having abnormal scores at all time points. In comparison, the OCS provided a comprehensive assessment across five domains. However, the OCS was difficult to summarise for comparison between groups that would make it less suitable for use in a full trial.

### Pilot trial clinical outcomes

There was only one detected ischaemic event on MRI in 50 postoperative scans, suggesting that it would perform poorly as an outcome measure and very large numbers would be required to show an effect, if one was present. No previous study has reported the presence of cerebral emboli on MRI following tourniquet use. Previous work has found 23% of patients undergoing total hip replacement surgery have evidence of cerebral emboli on MRI.^20^

Cognition was broadly similar at baseline between groups and got worse among participants at day 1 and then recovered at 1 week. In addition to potential cerebral emboli, other reasons for cognitive dysfunction following surgery include postoperative pain and type of anaesthesia used.^21 22^ However, there was a difference in the amount of missing cognitive data at day 1 compared with all other time points. The majority of this was within the group allocated to tourniquet inflated, where four participants felt too unwell to complete tests. This would make future comparisons of cognition at this time point in a trial challenging. The finding may be explained by the higher pain scores particularly at day 1 in this group. A smaller data set focused on just MoCA for cognitive assessment at this time point may be more acceptable than our much more comprehensive data set and have better completion. This could be considered in a future trial.

We found a trend towards patients having more thigh pain in those that had TKR with a tourniquet inflated, in keeping with previous published literature and our own Cochrane Systematic Review (under review).^23–25^ Higher levels of pain may lead to more patients requiring larger amounts of strong opiate based analgesia after surgery which may have is likely to have an effect on postoperative cognition.^13^

Both groups demonstrated worse OKS and EQ-5D-5L scores at 1 week post surgery. However, scores had improved from baseline by 6 months. This is in keeping with previous published research demonstrating improvements in knee pain and function and health-related quality of life 6 month to a year after surgery.^26^

The number of AEs related to surgery was higher in patients who had TKR with a tourniquet (21 vs 9), but the numbers in each type of AE were small. It is not possible to make robust conclusions, however, the finding of two DVTs in the group who had an inflated tourniquet is relevant because DVT is a rare event and it follows a similar trend across other published studies and our own Cochrane Review (not yet published) when data were pooled from multiple small studies.^3 25^

### Conclusions

The strengths of our pilot study include the study design and blinding of patients and assessors which should have minimised detection and performance bias. We have explored a wide range of important safety outcomes and demonstrate cognitive outcome measures which would be suitable for use in a full trial. The study was also only completed within one NHS trust, so there may be challenges in recruiting patients and collecting detailed outcome measures across multiple centres. However, TKR is performed in large numbers in the UK, and the screening data confirm that the study is acceptable to patients, so we are confident that it will be deliverable in a multicentre format although without the MRI component.

We designed the study to test feasibility. Hence, we have not made any statistical inferences; however, consistent trends in pain and AEs were found. This pilot trial has demonstrated recruitment to a full trial is feasible and has helped determine suitable primary outcomes (MoCA) and secondary outcomes (pain and AEs) that warrant investigation in a large multicentre trial.

## Patient and public involvement

A patient and public involvement (PPI) group was established prior to the study commencing. This group consisted of patient members who had recently undergone TKR surgery. The group met once a year and were instrumental in the study design. Recruitment strategies and outcome measures were discussed prior to selection. The final results were also discussed with the PPI group in order to ensure that an accurate and appropriate message was conveyed.

## References

[1] Registry NJ 16th annual report 2019, 2019 Available: https://reports.njrcentre.org.uk/Portals/0/PDFdownloads/NJR

[2] Scottish Arthroplasty Project Annual report, 2019.

[3] Zhang W, Li N, Chen S, et al The effects of a tourniquet used in total knee arthroplasty: a meta-analysis. J Orthop Surg Res 2014;9:13. 10.1186/1749-799X-9-13 24602486 PMC3973857

[4] National Joint Registry for England and Wales National joint Registry for England and Wales 1st annual report, 2004.

[5] Parvizi J, Diaz-Ledezma C Total knee replacement with the use of a tourniquet: more pros than cons. Bone Joint J 2013;95-B:133–4. 10.1302/0301-620X.95B11.32903 24187371

[6] Ejaz A, Laursen AC, Kappel A, et al Tourniquet induced ischemia and changes in metabolism during TKA: a randomized study using microdialysis. BMC Musculoskelet Disord 2015;16:326. 10.1186/s12891-015-0784-y 26510621 PMC4625433

[7] Tai T-W, Lin C-J, Jou I-M, et al Tourniquet use in total knee arthroplasty: a meta-analysis. Knee Surg Sports Traumatol Arthrosc 2011;19:1121–30. 10.1007/s00167-010-1342-7 21161177 PMC3116117

[8] Alcelik I, Pollock RD, Sukeik M, et al A comparison of outcomes with and without a tourniquet in total knee arthroplasty: a systematic review and meta-analysis of randomized controlled trials. J Arthroplasty 2012;27:331–40. 10.1016/j.arth.2011.04.046 21944371

[9] Senay A, Trottier M, Delisle J, et al Incidence of symptomatic venous thromboembolism in 2372 knee and hip replacement patients after discharge: data from a thromboprophylaxis registry in Montreal, Canada. Vasc Health Risk Manag 2018;14:81–9. 10.2147/VHRM.S150474 29780248 PMC5951148

[10] Park Y-G, Ha C-W, Lee SS, et al Incidence and Fate of "Symptomatic" Venous Thromboembolism After Knee Arthroplasty Without Pharmacologic Prophylaxis in an Asian Population. J Arthroplasty 2016;31:1072–7. 10.1016/j.arth.2015.11.028 26777576

[11] Berman AT, Parmet JL, Harding SP, et al Emboli observed with use of transesophageal echocardiography immediately after tourniquet release during total knee arthroplasty with cement. J Bone Joint Surg Am 1998;80:389–96. 10.2106/00004623-199803000-00012 9531207

[12] Sulek CA, Davies LK, Enneking FK, et al Cerebral microembolism diagnosed by transcranial Doppler during total knee arthroplasty: correlation with transesophageal echocardiography. Anesthesiology 1999;91:672–6. 10.1097/00000542-199909000-00018 10485777

[13] Deo H, West G, Butcher C, et al The prevalence of cognitive dysfunction after conventional and computer-assisted total knee replacement. Knee 2011;18:117–20. 10.1016/j.knee.2010.03.006 20615709

[14] Wall PD, Ahmed I, Metcalfe A, et al Safety and feasibility evaluation of tourniquets for total knee replacement (SAFE-TKR): study protocol. BMJ Open 2018;8:e022067. 10.1136/bmjopen-2018-022067 PMC5900804 29643169

[15] Fairbairn TA, Mather AN, Bijsterveld P, et al Diffusion-Weighted MRI determined cerebral embolic infarction following transcatheter aortic valve implantation: assessment of predictive risk factors and the relationship to subsequent health status. Heart 2012;98:18–23. 10.1136/heartjnl-2011-300065 21737581

[16] Ghanem A, Müller A, Nähle CP, et al Risk and fate of cerebral embolism after transfemoral aortic valve implantation: a prospective pilot study with diffusion-weighted magnetic resonance imaging. J Am Coll Cardiol 2010;55:1427–32. 10.1016/j.jacc.2009.12.026 20188503

[17] Gress DR The problem with asymptomatic cerebral embolic complications in vascular procedures: what if they are not asymptomatic? J Am Coll Cardiol 2012;60:1614–6. 10.1016/j.jacc.2012.06.037 22999732

[18] Lancaster GA, Dodd S, Williamson PR Design and analysis of pilot studies: recommendations for good practice. J Eval Clin Pract 2004;10:307–12. 10.1111/j.2002.384.doc.x 15189396

[19] Wall PDH, Parsons NR, Parsons H, et al A pragmatic randomised controlled trial comparing the efficacy of a femoral nerve block and periarticular infiltration for early pain relief following total knee arthroplasty. Bone Joint J 2017;99-B:904–11. 10.1302/0301-620X.99B7.BJJ-2016-0767.R2 28663395 PMC5633832

[20] Patel RV, Stygall J, Harrington J, et al Cerebral microembolization during primary total hip arthroplasty and neuropsychologic outcome: a pilot study. Clin Orthop Relat Res 2010;468:1621–9. 10.1007/s11999-009-1140-z 19838644 PMC2865620

[21] Hou R, Wang H, Chen L, et al POCD in patients receiving total knee replacement under deep vs light anesthesia: a randomized controlled trial. Brain Behav 2018;8:e00910. 10.1002/brb3.910 29484267 PMC5822567

[22] Zhang X, Xin X, Dong Y, et al Surgical incision-induced nociception causes cognitive impairment and reduction in synaptic NMDA receptor 2B in mice. J Neurosci 2013;33:17737–48. 10.1523/JNEUROSCI.2049-13.2013 24198365 PMC3818549

[23] Alexandersson M, Wang EY, Eriksson S A small difference in recovery between total knee arthroplasty with and without tourniquet use the first 3 months after surgery: a randomized controlled study. Knee Surg Sports Traumatol Arthrosc 2019;27:1035–42. 10.1007/s00167-018-5196-8 30328495 PMC6435610

[24] Kumar N, Yadav C, Singh S, et al Evaluation of pain in bilateral total knee replacement with and without tourniquet; a prospective randomized control trial. J Clin Orthop Trauma 2015;6:85–8. 10.1016/j.jcot.2015.01.095 25983513 PMC4411338

[25] Ahmed I, Chawla A, Underwood M, et al Tourniquet use for knee replacement surgery. Cochrane Database Syst Rev 2017;77:CD012874 10.1002/14651858.CD012874 PMC8094224 33316105

[26] Jawhar A, Skeirek D, Stetzelberger V, et al No effect of tourniquet in primary total knee arthroplasty on muscle strength, functional outcome, patient satisfaction and health status: a randomized clinical trial. Knee Surg Sports Traumatol Arthrosc 2020;28:1045–54. 10.1007/s00167-019-05646-5 31372679

