# Supplementary material for: Tourniquet use in total knee replacement surgery: a feasibility study and pilot randomised controlled trial (SAFE-TKR study)
Source: BMJ Open. 2021 Jan 22;11(1):e043564. doi: 10.1136/bmjopen-2020-043564 (PMC7825264; doi:10.1136/bmjopen-2020-043564)
Supplement: Supplementary data [file bmjopen-2020-043564supp001.pdf]

Supplementary file 1: Oxford Cognitive screen results.

Oxford Cognitive Screen Results

OCS scores within each cognitive domain are shown in supplementary table 1, there is no total summary score for this measure. However, across the five cognitive domains at baseline more participants had normal scores in the group allocated to tourniquet inflated versus tourniquet not inflated. Data was missing for six participants in the group allocated to tourniquet, four of these participants felt too unwell to complete the test, for one participant research staff were not available to administer it and for one participant the reason was not recorded. In comparison at day one data was missing for one participant in the group allocated to tourniquet not inflated, the reason for this missing data was not recorded. At day one in both groups there was a deterioration in the proportion of normal cognitive scores across all five domain, this improved at day two but not returned to baseline levels at one week.

Supplementary table 1: OCS Scores

Item	Baseline (n,%)		Day 1 (n,%)		Day 2 (n,%)		Week 1 (n,%)	
	T inflated	T not inflated	T inflated	T not inflated	T inflated	T not inflated	T inflated	T not inflated
Number randomised to group	27	26	27	26	27	26	27	26
Scores present (n, %t)	27 (100)	25 (96)	21 (78)	25 (96)	24 (89)	24 (92)	22 (81)	22 (85)
Numbers are of patients who record normal (not impaired) scores, percentages are of randomised group								
**1. Attention & executive function**								
Accuracy	26 (96)	22 (85)	19 (70)	17 (65)	21 (78)	22 (85)	20 (74)	19 (73)
Space neglect	24 (89)	22 (85)	15 (56)	19 (73)	21 (78)	22 (85)	19 (70)	17 (65)
Object neglect	25 (93)	21 (81)	20 (74)	24 (92)	23 (85)	23 (88)	20 (74)	20 (77)
Perseveration	26 (96)	23 (88)	21 (78)	25 (96)	23 (85)	23 (88)	21 (78)	20 (77)
Single rule	24 (89)	23 (88)	20 (74)	23 (88)	22 (81)	21 (81)	21 (78)	22 (85)
Alternating rule	25 (93)	20 (77)	18 (67)	21 (81)	20 (74)	20 (77)	20 (74)	21 (81)
Heart Organisation Index	27 (100)	22 (85)	19 (70)	23 (88)	20 (74)	21 (81)	20 (74)	19 (73)
**2. Language**								
Picture naming	26 (96)	25 (96)	21 (78)	24 (92)	23 (85)	23 (88)	21 (78)	22 (85)
Semantics	26 (96)	25 (96)	21 (78)	25 (96)	24 (89)	24 (92)	22 (81)	22 (85)
Sentence reading	24 (89)	22 (85)	14 (52)	20 (77)	19 (70)	20 (77)	21 (78)	19 (73)
**3. Memory**								
Overall verbal memory	26 (96)	24 (92)	21 (78)	23 (88)	24 (89)	24 (92)	22 (81)	22 (85)
Episodic Memory	27 (100)	24 (92)	20 (74)	22 (85)	22 (81)	24 (92)	21 (78)	22 (85)
Orientation	8 (30)	8 (31)	4 (15)	7 (27)	8 (30)	8 (31)	7 (26)	8 (31)
**4. Number processing**								
Numerical calculation	24 (89)	21 (81)	20 (74)	24 (92)	21 (78)	24 (92)	19 (70)	22 (85)
Number writing	24 (89)	20 (77)	18 (67)	20 (77)	19 (70)	21 (81)	17 (63)	20 (77)

**5. Praxis**								
Overall	22 (81)	16 (62)	16 (59)	17 (65)	22 (81)	18 (69)	12 (44)	15 (58)
**6. Visual fields**								
Visual Perception: Total correct	22 (81)	24 (92)	14 (52)	18 (69)	18 (67)	23 (88)	16 (59)	22 (85)
Visual field	20 (74)	23 (88)	15 (56)	19 (73)	18 (67)	22 (85)	19 (70)	22 (85)
Visual Extinction: Left	26 (96)	23 (88)	20 (74)	22 (85)	22 (81)	24 (92)	20 (74)	22 (85)
Visual Extinction: Right	25 (93)	24 (92)	19 (70)	22 (85)	23 (85)	23 (88)	20 (74)	22 (85)
